# Immunonutritive Scoring for Patients with Hepatocellular Carcinoma Undergoing Transarterial Chemoembolization: Evaluation of the CALLY Index

**DOI:** 10.3390/cancers13195018

**Published:** 2021-10-07

**Authors:** Lukas Müller, Felix Hahn, Aline Mähringer-Kunz, Fabian Stoehr, Simon Johannes Gairing, Maurice Michel, Friedrich Foerster, Arndt Weinmann, Peter Robert Galle, Jens Mittler, Daniel Pinto dos Santos, Michael Bernhard Pitton, Christoph Düber, Roman Kloeckner

**Affiliations:** 1 Department of Diagnostic and Interventional Radiology, University Medical Center of the Johannes Gutenberg University Mainz, 55131 Mainz, Germany; lukas.mueller@unimedizin-mainz.de (L.M.); felix.hahn@unimedizin-mainz.de (F.H.); aline.maehringer-kunz@unimedizin-mainz.de (A.M.-K.); fabian.stoehr@unimedizin-mainz.de (F.S.); michael.pitton@unimedizin-mainz.de (M.B.P.); christoph.dueber@unimedizin-mainz.de (C.D.); 2 Department of Internal Medicine, University Medical Center of the Johannes Gutenberg University Mainz, 55131 Mainz, Germany; simonjohannes.gairing@unimedizin-mainz.de (S.J.G.); maurice.michel@unimedizin-mainz.de (M.M.); friedrich.foerster@unimedizin-mainz.de (F.F.); arndt.weinmann@unimedizin-mainz.de (A.W.); peter.galle@unimedizin-mainz.de (P.R.G.); 3 Department of General, Visceral and Transplant Surgery, University Medical Center of the Johannes Gutenberg University Mainz, 55131 Mainz, Germany; jens.mittler@unimedizin-mainz.de; 4 Department of Radiology, University Hospital of Cologne, 50937 Cologne, Germany; daniel.pinto-dos-santos@uk-koeln.de

**Keywords:** hepatocellular carcinoma, transarterial chemoembolization, risk prediction, inflammation index, immunoscoring, CRP–albumin–lymphocyte ratio

## Abstract

**Simple Summary:**

The novel CRP–albumin–lymphocyte (CALLY) index has been identified as a highly predictive tool for stratification of patients with hepatocellular carcinoma (HCC) who have undergone tumor resection. This study aimed to validate the predictive ability of the CALLY index in patients with HCC treated with transarterial chemoembolization (TACE). The CALLY index was an independent prognostic predictor for overall survival. However, the CALLY index was not superior to other immunonutritive and inflammation scoring systems in predicting the median OS, although all of the individual parameters of the CALLY index were predictive for the median OS. Thus, future studies should re-evaluate the mathematical calculation of the index for patients with HCC undergoing TACE.

**Abstract:**

The novel CRP–albumin–lymphocyte (CALLY) index is an improved immunonutritive scoring system, based on serum C-reactive protein (CRP), serum albumin, and the lymphocyte count. It has shown promise as a prognostic index for patients with hepatocellular carcinoma (HCC) undergoing resections. This study evaluated the prognostic ability of the CALLY index for patients with HCC undergoing transarterial chemoembolization (TACE). We retrospectively identified 280 treatment-naïve patients with HCC that underwent an initial TACE at our institution, between 2010 and 2020. We compared the CALLY index to established risk factors in univariate and multivariate regression analyses for associations with median overall survival (OS). A low CALLY score was associated with low median OS (low vs. high CALLY: 9.0 vs. 24.0 months, *p* < 0.001). In the multivariate analysis, the CALLY index remained an independent prognostic predictor (*p* = 0.008). Furthermore, all factors of the CALLY index reached significance in univariate and in-depth multivariate analyses. However, the concordance index (C-index) of the CALLY index (0.60) was similar to the C-indices of established immunonutritive and inflammation scoring systems (range: 0.54 to 0.63). In conclusion, the CALLY index showed promise as a stratification tool for patients with HCC undergoing TACE. Notably, the CALLY index was not superior to other immunonutritive and inflammation scoring systems in predicting the median OS. Thus, future studies should re-evaluate the mathematical calculation of the index, particularly the contributions of individual parameters.

## 1. Introduction

Hepatocellular carcinoma (HCC) is the most common primary liver cancer, and it ranks among the deadliest cancer entities worldwide [1,2]. The Barcelona Clinic Liver Cancer (BCLC) classification system was recommended by the Western guidelines of the European Association for the Study of the Liver (EASL) and the American Association for the Study of Liver Diseases as a stratification system for treatment allocations and survival outcome estimations [3,4]. According to the BCLC classification, transarterial chemoembolization (TACE) is the standard of care for patients within intermediate-stage HCC [5,6]. In clinical practice, patients in the intermediate stage of HCC comprise a heterogeneous group, with broad variations in remnant liver function and tumor burden [7,8]. Thus, it remains difficult to predict the prognosis and make treatment decisions for these patients. To date, several scoring systems have been proposed to facilitate decision making [9,10,11]. However, all of these scores have shown a lack of reproducibility in external validation studies, which limits their integration into the routine clinical workflow [12,13]. 

Conventional risk models mainly consist of tumor burden and remnant liver function. In the future, these models could be refined. Increases in our knowledge about inflammation and immune system reactions have suggested that these parameters might carry additional prognostic information [14].

The PNI was originally developed in 1980 for patients undergoing gastrointestinal surgery [15]. Since then, the PNI has been identified as a highly predictive index for several cancer entities [16]. The PNI is a combination of the blood albumin level, which indicates the remnant liver function, and the blood lymphocyte count, which indicates the immune system status. In a recent study, we showed that the PNI was a favorable immunonutritive scoring system for survival stratification in patients with HCC that were undergoing TACE [17]. 

A previous study identified C-reactive protein (CRP) as a prognostic factor for patients with HCC [18]. We hypothesized that CRP might serve as an additional component for immunonutritive scoring because it could reflect the inflammatory component of the tumor microenvironment. Thus, we developed the novel CRP–albumin–lymphocyte (CALLY) index, which combines the PNI with the CRP level. We reasoned that this unique combination of markers for liver function, immune system status, and inflammation might have a synergistic effect in predicting survival in patients with HCC. Indeed, the CALLY index showed promising predictive ability in patients with early HCC that were undergoing a resection [19]. Therefore, a priori, we expected that the CALLY index would show high predictive ability for patients with HCC undergoing TACE. To the best of our knowledge, no previous study has evaluated the CALLY index in patients with HCC that underwent TACE. Furthermore, no direct head-to-head comparison between the CALLY index and the established PNI has been performed.

This study aimed to investigate whether the addition of CRP to existing immune-based prognostic indices might improve the stratification of patients with HCC undergoing TACE. Therefore, the novel CALLY index was evaluated extensively and compared head to head to an immunonutritive index (i.e., the PNI) and to other “purely” immune-based indices.

## 2. Materials and Methods

This analysis of clinical data was approved by the Ethics Committee of the Medical Association of Rhineland Palatinate, Mainz, Germany (permit number 2021-15666). The requirement for informed consent was waived, due to the retrospective nature of the study. Patient records and information were anonymized and de-identified prior to analysis. TRIPOD guidelines were followed for drafting this manuscript.

### 2.1. Patients

**Figure 1:**
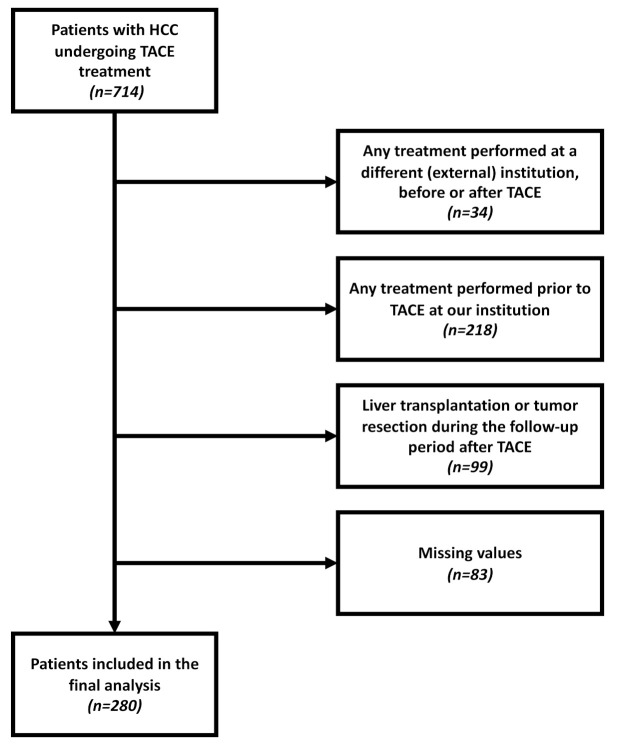
Flowchart showing the various reasons why patients were excluded from this study.

A total of 714 patients with confirmed HCC were referred to our tertiary care center for TACE treatment between January 2010 and November 2020. Among these patients, 434 were excluded from the study for the reasons shown in Figure 1. Thus, 280 treatment-naïve patients with complete laboratory and imaging data were included in the final analysis. In addition, a subgroup analysis was conducted with 154 (55.0%) patients classified as BCLC stage B; in this stage, TACE is the recommended first-line therapy according to current recommendations [3,20].

### 2.2. Diagnosis, Treatment, and Follow-Up

As previously reported, histological or image-based EASL criteria were used to diagnose HCC [3,17]. Prior to their first TACE treatment, all patients underwent contrast-enhanced computed tomography (CT) or magnetic resonance imaging (MRI) to inform the diagnosis, staging, and treatment planning. These data were extensively discussed in an interdisciplinary tumor board that included hepatologists/oncologists, diagnostic and interventional radiologists, visceral surgeons, pathologists, and radiation therapists. 

TACE was performed in a standardized manner, as described in detail previously [21,22]. The follow-up examinations included a clinical examination, blood sample analysis, and cross-sectional imaging. In patients with a viable tumor, imaging was typically repeated every 6 weeks. When the imaging results showed a complete response, the interval was extended to 12 weeks. The primary study endpoint was the median overall survival (OS), defined as the time interval between the initial TACE treatment and death or last follow-up. 

### 2.3. Data Acquisition

The dataset was acquired from the clinical registry unit (CRU), as previously reported [17]. The CRU is an established registry that prospectively collects data on all patients with liver cancer treated at our tertiary referral center [23]. For the present analysis, we retrieved data from the CRU dataset on all baseline characteristics, including demographic data; liver disease status and etiology; laboratory parameters; TACE-related parameters; and information on tumor burden, including the tumor growth pattern, number of lesions, and the diameter of the largest target lesion. When necessary, the CRU dataset was completed with data from the radiology information system, the hospital information system, and the laboratory database. 

### 2.4. Calculation of the CALLY Index

**Figure 2:**
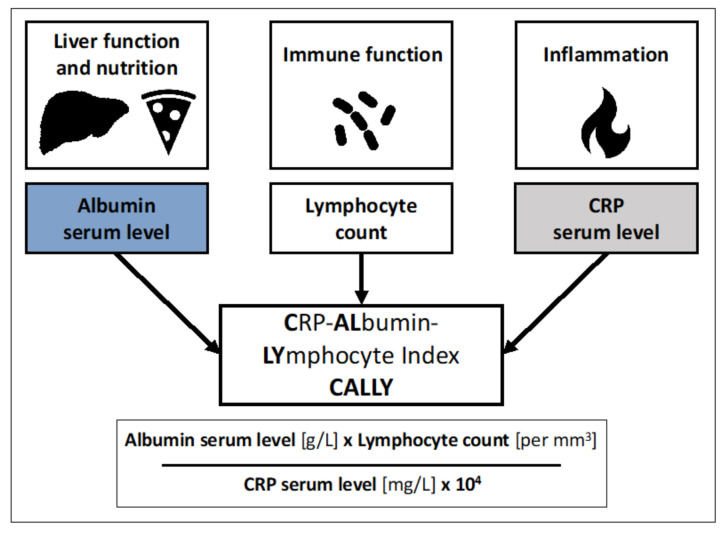
Overview of the formula for calculating the CRP–albumin–lymphocyte (CALLY) index.

The CALLY index was calculated as described previously [19]. Figure 2 presents an overview of the calculation of the CALLY index. We also calculated several other scoring systems, including the neutrophil-to-lymphocyte ratio (NLR), the platelet-to-lymphocyte ratio (PLR), the systemic immune-inflammation index (SII), the integrated liver inflammatory score (ILIS), and the PNI, as previously described [24,25,26,27,28].

### 2.5. Statistical Analysis

Statistical analyses and graphic designs were performed in R 4.0.3 (A Language and Environment for Statistical Computing, R Foundation for Statistical Computing, http://www.R-project.org; last accessed 15 August 2021). Continuous data are reported as the median and range. Categorical and binary baseline parameters are reported as absolute numbers and percentages. Standardized cut-off values for the laboratory parameters were derived from our laboratory database. Optimal stratification was used to calculate the cut-off values for the immunonutritive and inflammation indices, including the CALLY index, with software packages “survminer” and “survival” (https://cran.r-project.org/package=survminer, https://CRAN.R-project.org/packagurvival, last accessed 15 August 2021). Additionally, we used the same software packages to perform survival analyses, including the Kaplan–Meier curves. The different strata of these curves were compared with log-rank testing. The “Number at risk” was defined as the number of patients with HCC undergoing TACE that remained alive at the various timepoints. Hazard ratios (HRs) and corresponding 95% confidence intervals (CIs) were assessed with univariate and multivariate Cox proportional hazard regression models. Further comparisons between the CALLY index and the existing immunonutritive and inflammation indices were performed with Harrell’s C concordance index (C-index) with the “Hmisc” package (https://cran.r-project.org/package=Hmisc, last accessed 15 August 2021). A C-index of 1.0 indicated perfect predictive power, and a C-index of 0.5 indicated no predictive ability [29]. Values of *p* < 0.05 were considered statistically significant for all tests.

## 3. Results

### 3.1. Baseline Characteristics

Patient baseline characteristics are presented in Table 1. A subgroup analysis was performed on 154 (55.0%) patients classified as BCLC stage B (i.e., the subgroup for which TACE was recommended).

### 3.2. Survival Analysis 

**Figure 3:**
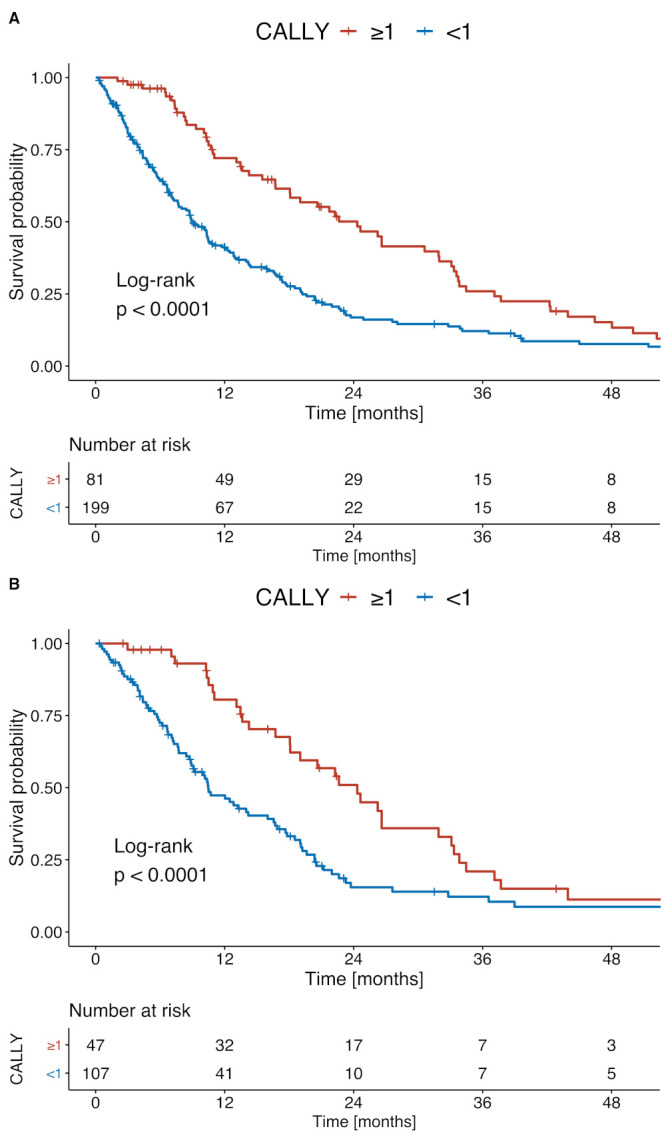
Kaplan–Meier survival curves comparing patients with low and high CALLY scores among (**A**) all patients and (**B**) patients classified as BCLC B.

After applying optimal stratification, the optimal cut-off for the median OS was 1 point for the CALLY index. With this cut-off value, 199 (71.1%) patients had a low CALLY score and 81 (28.9%) had a high CALLY score. The corresponding median OS values for the low and high CALLY groups were 9.0 months and 24.0 months (*p* < 0.001, Figure 3A), respectively. For the subgroup of patients classified as BCLC stage B, the optimal stratification yielded the same optimal cut-off of 1 point for the median OS. Thus, 107 (69.5%) patients had a low CALLY score and 47 (30.5%) patients had a high CALLY score. The corresponding median OS values for the low and high CALLY groups were 13.1 months and 26.2 months (*p* < 0.001, Figure 3B), respectively.

In the univariate Cox hazard regression analysis, the CALLY index showed high prognostic value. Other factors with high prognostic value were the bilirubin level, the AST level, and the tumor number. In the subsequent multivariate analysis, which included all of the above-mentioned significant factors, only a low CALLY score, a high bilirubin level, and a high AST level remained significant predictors (Table 2).

In the subgroup of patients classified as BCLC stage B, the CALLY score, the bilirubin level, and the tumor lesion size showed high predictive value in the univariate Cox hazard regression. In the multivariate Cox hazard regression, only a low CALLY score and a high bilirubin level remained significant risk factors (Table 3).

In a separate, in-depth analysis of the individual CALLY parameters, all factors were highly predictive of the median OS in univariate and multivariate analyses, when optimal stratification was applied. Similarly, in the analysis of the subgroup classified as BLCL B, all the individual CALLY factors reached significance in both univariate and multivariate analyses (Table 4).

When we compared the CALLY index to other established immunonutritive and inflammation scoring systems (i.e., the NLR, PLR, PNI, SII, and ILIS), we found that the C-index of the CALLY index was slightly lower than the C-index of the PNI and slightly higher than the C-indices of the other scores (Table 5). This tendency was similar when we performed this comparison in the subgroup of patients classified as BLCL B (Table 5). 

## 4. Discussion

To the best of our knowledge, this study is the first to investigate the role of the CALLY index for patients with HCC that underwent TACE. The CALLY index could stratify patients according to the median OS, and it remained an independent prognostic factor in multivariate analyses for all patients, including the subgroup of patients classified as BLCL B. In addition, each individual parameter of the CALLY index, namely, the serum CRP level, the serum albumin level, and the lymphocyte count, was highly predictive of the median OS. Nevertheless, the C-index of the CALLY index was not superior to the C-indices of the other investigated immunonutritive and inflammation scoring systems.

In more than 80% of cases, HCC develops from liver cirrhosis [3]. In the injured milieu, inflammatory processes and counter-regulation have been identified as the key drivers of HCC development and progression [30,31]. Lymphocytes act as counter-regulators to non-resolving inflammation and transformation due to unregulated proliferation. Indeed, lymphocytes are an important part of the host tumor defense mechanism because they inhibit cell proliferation and migration [30,32]. 

Immune-based scoring has been widely investigated for patients with HCC [25,26]. Indeed, several recent studies have investigated the prognostic roles of various indices [33,34]. In particular, the NLR and PLR have been investigated extensively as prognostic indices for patients with HCC undergoing TACE [33,35]. However, the prognostic ability of both those indices varied in several validation studies. The SII is based on the factors included in the NLR and PLR indices, and it was recently validated as a potential novel scoring system for patients with HCC undergoing TACE [34]. Nevertheless, the predictive ability of the SII was not superior to that of the NLR and PLR, consistent with our findings in the present study. Additionally, the ILIS was recently promoted as an immune-based score specifically for patients with HCC. The first validation study, conducted in patients undergoing TACE, demonstrated a C-index of 0.625, which was higher than the C-index found in our dataset. Nevertheless, future large-scale studies on both the SII and the ILIS are mandatory to evaluate their prognostic predictive abilities, compared to existing immune-based indices. In contrast to immune-based indices, immunonutritive indices integrate the nutritional status into the prognostic score. Theoretically, the immunonutritional status should lead to a more integrated patient evaluation. Accordingly, in a recent study, we demonstrated that the PNI showed promise as a prognostic indicator for patients with HCC undergoing TACE [17]. However, studies on the immunonutritional status are scarce for this patient group, and future large-scale studies are needed. Table 6 summarizes the characteristics, concepts, and the pros and cons of the various immunonutritive and immune-based indices, with notes on current and future research strategies.

Several conventional tools and scoring systems have been developed to provide support for clinicians making treatment decisions for patients with HCC that undergo TACE [39]. Unfortunately, despite promising initial results, all the conventional risk scores have failed in external evaluations [9,10,11,12,13]. All these scores mainly include tumor size, tumor number, and alpha fetoprotein as surrogates for the tumor burden and remnant liver function. Historically, liver function in patients with HCC is assessed with the Child–Pugh score. This score combines albumin, the international normalized ratio (INR), bilirubin, ascites, and hepatic encephalopathy [40]. However, estimations of ascites and hepatic encephalopathy rely on subjective interpretations; therefore, more quantitative assessment systems have been validated for patients with HCC undergoing TACE [41,42,43]. These quantitative systems are based on laboratory parameters such as the albumin-bilirubin (ALBI) grade, the Model of End Stage Liver Disease (MELD) score, and the MELD-Natrium score. Nevertheless, liver function assessments have shown variable prognostic abilities. Furthermore, liver function assessment is only one of several assessments considered in making treatment decisions for patients with HCC that undergo TACE. Thus, additional aspects of patients’ condition must be considered for a more holistic patient evaluation.

Overall, none of the established risk scores or liver function estimates include recent insights on how inflammation, the immune response, and nutrition affect HCC development and progression. Thus, immunonutritive indices have the potential to reflect more aspects of cancer development and progression. Accordingly, these indices could improve patient stratification and enable more individualized prognosis predictions. Inflammation and immunonutritive indices will not replace liver function and tumor burden assessments in treatment decision making; however, they could provide a more holistic picture of the patient’s overall status.

In this study, we first investigated the role of the novel CALLY index in predicting prognosis in patients with HCC undergoing TACE. Compared to the above-mentioned existing indices, the CALLY index combines markers of inflammation, the immune response, and nutritional status. Based on the recent insights into the prognostic roles of immunonutrition and CRP for patients with HCC undergoing TACE, the combination of these markers and their evaluation seemed to be the next step for advancing the field [18,44]. Our univariate and multivariate analyses confirmed that including all these factors in the CALLY index showed promising results. Interestingly, the CALLY index showed only slightly superior predictive ability compared to the established immunonutritive and immune-based scoring systems. Moreover, the CALLY index was slightly inferior to the PNI in predicting the median OS of patients with HCC undergoing TACE. Consequently, we conclude that the combination of inflammation, immune response, and nutritional status markers has the potential of improving immunonutritive scoring; thus, the CALLY index should be integrated into the above-mentioned existing stratification systems, as an additional component of holistic patient evaluations. However, the CALLY index calculation might not be optimal for patients with HCC undergoing TACE. Future studies should re-evaluate the mathematical calculation of the CALLY index to optimize the contributions of the individual parameters. Additionally, large-scale prospective trials could facilitate the identification of optimal cut-off values for further refining the CALLY index.

To date, because the CALLY index is a novel prediction system, we lack reference values. Thus, the CALLY index equation might be only partly suitable for patients with HCC undergoing TACE. Future studies should re-evaluate the mathematical calculation of the CALLY index, particularly the contributions of the individual parameters. Additionally, large-scale prospective trials could facilitate the identification of optimal cut-off values that can be used to refine the CALLY index further.

Our multivariate analysis confirmed that bilirubin was a highly predictive factor for the entire patient cohort, and for the subgroup of patients classified as BLCL B. Thus, the predictive ability of the CALLY index might be further improved if it were combined with the bilirubin level. Indeed, the albumin-bilirubin (ALBI) grade is a well-established scoring system for evaluating remnant liver function [41,42]. A previous study showed that the combination of the PNI, an immunonutritive scoring system, and the ALBI grade was a promising stratification tool for evaluating patients with HCC for remnant liver function and immunonutritive status [45]. In a recent study, we showed that the so-called ALBI-PNI was feasible, particularly among patients undergoing TACE [46]. Thus, our future research effort could include the integration of CRP as a relevant prognostic factor in the ALBI-PNI system.

In the future, immunonutritive scoring could be combined with imaging assessments of body composition parameters. In particular, the estimation of sarcopenia, defined as the quantitative and qualitative loss of skeletal muscle mass, has been identified as a highly predictive factor for patients with HCC [47,48]. In combination with the promising results on immunonutritive scoring, imaging might further enhance appraisals of patient status and improve stratification and scoring systems.

This study had several limitations. First, it was a single-center study. Second, the sample size was only moderate (*n* = 280). However, we only included patients with complete datasets because we actively decided against imputing missing values. Furthermore, we only included patients treated from 2010 and later to ensure comparability in the diagnosis, treatment, and follow-up procedures. In addition, to avoid bias, we excluded patients that underwent subsequent liver transplantation or other curative treatments after TACE [43]. Nevertheless, compared to previous studies on this issue, our sample size was comparable [19].

## 5. Conclusions

We found that the novel CALLY index was a promising stratification tool for patients with HCC undergoing TACE. Notably, although all three individual parameters were predictive of the median OS, the predictive ability of the CALLY index was not superior to the abilities of other immunonutritive and inflammation scoring systems. Thus, future large-scale studies should re-evaluate the mathematical calculation of the CALLY index, particularly the contributions of the individual parameters.

## Figures and Tables

**Table 1:** Baseline characteristics of patients with HCC undergoing TACE included in this study.

Variable	All Patients (*n* = 280)	Patients Classified as BCLC B (*n* = 154)
Age, years	69.5 (62.5–75.4)	70.2 (63.3–75.6)
Sex, *n* (%)		
Female	46 (16.4)	23 (14.9)
Male	234 (83.6)	131 (85.1)
Etiology, *n* (%)		
Alcohol	131 (46.8)	70 (45.5)
Hepatitis C	46 (16.4)	25 (16.2)
Hepatitis B	26 (9.3)	17 (11.0)
NASH	27 (9.6)	15 (9.7)
Hemochromatosis	5 (1.8)	3 (2.0)
AIH/PBC/PSC	5 (1.8)	3 (2.0)
Unknown/Other	29 (10.4)	13 (8.4)
None	11 (3.9)	8 (5.2)
Child–Pugh stage, *n* (%)		
A	104 (37.2)	60 (39.0)
B	116 (41.4)	74 (48.0)
C	25 (8.9)	0
No cirrhosis	35 (12.5)	20 (13.0)
BCLC stage, *n* (%)		
0	0	0
A	45 (16.1)	0
B	154 (55.0)	154 (100)
C	58 (20.7)	0
D	23 (8.2)	0
Max. tumor size, cm	4.2 (2.9–6.4)	4.3 (3.2–6.1)
Tumor number, *n* (%)		
Unifocal	55 (19.6)	0
Multifocal	203 (72.5)	146 (94.8)
Diffuse growth pattern	22 (7.9)	8 (5.2)
Albumin level, g/L	31 (27–35)	31 (28–35)
Lymphocyte count, per mm^3^	1214 (83–1558)	1263 (841–1660)
Bilirubin level, mg/dL	1.3 (0.8–2.2)	1.3 (0.9–2.0)
Platelet count, per nL	128 (87–193)	119 (84–194)
AST level, U/L	64.5 (47.0–95.5)	63.0 (47.0–88.5)
ALT level, U/L	41.5 (28.0–61.0)	42.0 (28.0–62.0)
AP level, U/L	156.0 (114.0–212.3)	146.5 (104.0–201.8)
CRP level, mg/L	9.0 (3.6–18.0)	8.0 (3.6–15.8)
INR	1.2 (1.1–1.3)	1.1 (1.0–1.3)
AFP level, ng/mL	45.0 (8.1–777.0)	48.0 (7.6–593.5)

Values are given as *n* (%) or median (interquartile range) unless otherwise noted. NASH, nonalcoholic steatohepatitis. AIH, autoimmune hepatitis. PBC, primary biliary cholangitis. PSC, primary sclerosing cholangitis. BCLC, Barcelona Clinic Liver Cancer. AST, aspartate aminotransferase. ALT, alanine aminotransferase. AFP, alpha fetoprotein.

**Table 2:** Univariate and multivariate analysis results for all patients.

Analysis		Univariate			Multivariate		
Covariate	Cut-Off	HR	95% CI	*p*-Value	HR	95% CI	*p*-Value
CALLY	<1 point	1.9	1.4–2.6	<0.001	1.5	1.1–2.1	0.008
Age	≥70 years	1.0	0.8–1.3	0.960			
AFP	>400 ng/mL	0.9	0.7–1.2	0.620			
Bilirubin level	≥1.2 mg/dL	2.1	1.6–2.7	<0.001	1.8	1.4–2.5	<0.001
AST level	>31 U/L	2.0	1.1–3.7	0.025	2.0	1.1–3.6	0.033
ALT level	≥35 U/L	1.2	0.9–1.6	0.200			
INR level	>1.2	1.1	0.8–1.5	0.460			
Platelet count	>150/nL	1.3	0.9–1.7	0.140			
Tumor number	≥2	1.5	1.0–2.1	0.027	1.2	0.8–1.6	0.388
Max. lesion size	>5.0 cm	1.3	1.0–1.7	0.058			

AFP, alpha fetoprotein. AST, aspartate aminotransferase. ALT, alanine aminotransferase.

**Table 3:** Univariate and multivariate analysis results for patients classified as BCLC B.

Analysis		Univariate			Multivariate		
Covariate	Cut-off	HR	95% CI	*p*-Value	HR	95% CI	*p*-Value
CALLY	<1 point	1.9	1.3–2.9	0.002	1.5	1.1–2.1	0.010
Age	≥70 years	1.0	0.7–1.5	0.930			
AFP	>400 ng/mL	1.0	0.7–1.4	0.790			
Bilirubin level	≥1.2 mg/dL	1.9	1.3–2.8	<0.001	1.9	1.4–2.5	<0.001
AST level	>31 U/L	1.5	0.7–2.9	0.280			
ALT level	≥35 U/L	1.3	0.9–1.9	0.230			
INR level	>1.2	1.1	0.7–1.6	0.770			
Platelet count	>150/nL	1.1	0.7–1.7	0.720			
Max. lesion size	>5.0 cm	1.5	1.0–2.3	0.035	1.3	1.0–1.7	0.069

AFP, alpha fetoprotein. AST, aspartate aminotransferase. ALT, alanine aminotransferase.

**Table 4:** Univariate and multivariate Cox proportional hazard regression model results for evaluating the individual CALLY parameters.

Analysis	Univariate			Multivariate		
Covariate	HR	95% CI	*p*-Value	HR	95% CI	*p*-Value
All patients (*n* = 280)						
Serum albumin level ≤ 31 g/L	2.5	1.9–3.3	<0.001	1.9	1.4–2.6	<0.001
Total lymphocyte count ≤ 647.9/mm^3^	1.7	1.2–2.5	0.005	1.5	1.0–2.2	0.036
Serum CRP level ≤ 12 mg/L	3.7	2.8–5.0	<0.001	3.0	2.2–4.1	<0.001
BCLC B subgroup (n = 164)						
Serum albumin level ≤29 g/L	2.6	1.7–3.8	<0.001	2.4	1.6–3.6	<0.001
Total lymphocyte count ≤ 727/mm^3^	2.2	1.4–3.5	<0.001	1.7	1.0–2.7	0.038
Serum CRP level ≤ 4 mg/L	*2.0*	*1.3–3.1*	<0.001	*2.0*	*1.3–3.0*	*0.001*

CRP, C-reactive protein. HR, hazard ratio. CI, confidence interval.

**Table 5:** Comparison of the CALLY index to existing immunonutritive and inflammation indices.

Score		Median OS	HR	95% CI	*p*-Value	C-Index
All Patients (*n* = 280)						
CALLY	≥1	24.0	Reference			0.60
	<1	9.0	1.9	1.4–2.6	<0.001	
NLR	≤3	16.5	Reference			0.59
	>3	7.6	1.8	1.4–2.4	<0.001	
PLR	<164	12.8	Reference			0.54
	≥164	10.2	1.3	1.0–1.8	0.082	
PNI	>36	18.8	Reference			0.63
	≤36	6.5	2.4	1.8–3.2	<0.001	
SII	<664	13.8	Reference			0.56
	≥664	8.6	1.5	1.1–2.1	0.006	
ILIS	<11	18.8	Reference			0.57
	≥11	10.2	1.5	1.3–2.5	<0.001	
BLCL B subgroup (*n* = 164)						
CALLY	≥1	26.2	Reference			0.61
	<1	13.1	1.9	1.3–2.9	0.002	
NLR	≤3	18.3	Reference			0.59
	>3	9.0	2.5	1.6–3.8	<0.001	
PLR	<214	16.5	Reference			0.55
	≥214	7.5	2.5	1.4–4.4	0.002	
PNI	>39	20.9	Reference			0.63
	≤39	10.3	1.9	1.3–2.8	<0.001	
SII	<622	16.7	Reference			0.55
	≥622	10.0	1.9	1.2–3.1	0.007	
ILIS	<31	17.9	Reference			0.58
	≥31	10.4	2.0	1.4–3.0	<0.001	

NLR, neutrophil-to-lymphocyte ratio. PLR, platelet-to-lymphocyte ratio. SII, systemic immune-inflammation index. ILIS, integrated liver inflammatory score. PNI, prognostic nutritional index.

**Table 6:** Overview of various aspects of the included immunonutritive and immune-based indices.

Index	Concept and Characteristics	Included Parameters	Pros	Cons	Current Research Status	Future Research Strategies
Immunonutritive Indices						
CALLY	- combines inflammation, immune response, and nutritional status markers (aspects of the PNI) - for liver disease, albumin functions as an indicator of liver function	- CRP - albumin - lymphocyte count	- novel combination of inflammation, immune response, nutritional status, and liver function markers provides a more holistic assessment	- scarce literature available - in this study, CALLY was not superior to previously established scoring systems	- designed for a cohort of patients with HCC undergoing resections [19] - only validated in this study for patients with HCC undergoing TACE	- further validation needed for patients with HCC undergoing TACE - validation for assessing other HCC treatment options - efficacy of CALLY combined with bilirubin - the mathematical calculation may require improvement
PNI	- combines immune response and nutritional status markers	- albumin - lymphocyte count	- combination of immune response and nutritional status markers	- few studies available on patients with HCC undergoing TACE - divergent results regarding the predictive ability of PNI - the mathematical calculation may require improvement	- designed for patients with gastric cancer [15] - extensively validated for various cancer entities - few studies available for patients with HCC undergoing TACE - divergent results on its predictive ability - PNI combined with ALBI was identified as a novel, feasible stratification system for patients with HCC undergoing TACE	- further, large-scale validation needed for patients with HCC undergoing TACE - the integration of immunonutrition into existing stratification systems
Other Immune-Based Indices						
NLR	- captures shifts in the relationships between blood cells, due to immune response effects	- neutrophil count - lymphocyte count	- simple calculation - well investigated	- nutritional status not included - divergent results in studies that compared NLR to other immune-based indices	- designed for the stratification of critically ill patients, and validated in patients with colorectal cancer, in an oncologic context [36] - extensively validated for various cancer entities, including patients with HCC undergoing TACE	- NLR combined with nutritional status markers - NLR combined with liver function markers - large-scale validation for patients with HCC undergoing TACE - integration into existing stratification tools
PLR	- captures shifts in the relationships between blood cells, due to immune response effects	- platelet count - lymphocyte count	- simple calculation - well investigated	- nutritional status not included - divergent results in studies that compared PLR to other immune-based indices	- designed for the stratification of patients with pancreatic cancer [37] - extensively validated for patients with HCC undergoing TACE	- PLR combined with nutritional status markers - PLR combined with liver function markers - further large-scale validation for patients with HCC undergoing TACE - integration into existing stratification tools
SII	- combines inflammation and immune response markers	- lymphocyte count - neutrophil count - platelet count	- extensively validated for patients with HCC	- nutritional status not included - literature is scarce for patients undergoing TACE	- designed for the stratification of patients with HCC undergoing resections [38] - extensively validated for various cancer entities - few studies on the role of the SII in patients undergoing TACE	- SII combined with nutritional status markers - SII combined with liver function markers - further large-scale validation for patients with HCC undergoing TACE - integration into existing stratification tools
ILIS	- combines inflammation, liver function, and tumor markers - specifically developed for patients with HCC	- albumin - bilirubin - alkaline phosphatase - neutrophil count	- index is specific for HCC - includes tumor and liver function markers	- complex calculation - scarce literature for patients with HCC, particularly for patients undergoing TACE	- specifically designed for patients with HCC [27] - only one external validation study available	- large-scale validation is mandatory - integration into existing stratification tools

## Data Availability

Data cannot be shared publicly because of institutional and national data policy restrictions imposed by the Ethics Committee of the Medical Association of Rhineland Palatinate, Mainz, Germany, since the data contain potentially identifying patient information. Data are available upon request for researchers who meet the criteria for access to confidential data.
